# Correlation between hippocampal volumes and proton magnetic resonance spectroscopy of the posterior cingulate gyrus and hippocampi in Alzheimer’s disease

**DOI:** 10.1590/S1980-57642010DN40200006

**Published:** 2010

**Authors:** Eun Joo Park, Katarina P. Lyra, Hae Won Lee, Paulo Caramelli, Maria C. G. Otaduy, Claudia Costa Leite

**Affiliations:** 1 Department of Radiology, School of Medicine of the University of São Paulo, São Paulo SP, Brazil.; 2 Faculty of Medicine, Federal University of Minas Gerais, Belo Horizonte MG, Brazil.

**Keywords:** hippocampal volumetry, proton magnetic resonance spectroscopy, Alzheimer’s disease

## Abstract

Prior studies have reported hippocampal volume loss, decrease in N-Acetylaspartate (NAA) concentration and increased myo-inositol (mI) concentration in patients with Alzheimer’s disease (AD). The purpose of this study was to evaluate hippocampal volumes of AD patients and their correlation with metabolic changes detected by proton spectroscopy (1H MRS) of hippocampal formations and the posterior cingulate region.

**Materials and Methods:**

22 patients with probable AD (18 mild, 4 moderate) and 14 elderly controls without cognitive symptoms, were enrolled in the study. Hippocampal volumetric measurements, single-voxel 1H MRS of the posterior cingulate region and of hippocampal formations were obtained. The following metabolite ratios were evaluated: NAA/Cr, mI/Cr, mI/NAA. Statistical analysis was performed to detect differences and correlations between these parameters in patients and controls.

**Results:**

The hippocampal volume of patients and controls did not differ significantly. The results of 1H MRS differed significantly between patients and controls in the hippocampal formations (mI/Cr, mI/NAA) and posterior cingulate region (NAA/Cr, mI/Cr, mI/NAA). The best predictor of AD diagnosis was NAA/Cr in the posterior cingulate region, having a sensitivity of 0.899 and specificity of 0.800. There was no correlation between hippocampal volumes and the results of 1H MRS in patients with AD.

**Conclusions:**

The results of 1H MRS differed significantly between patients and controls in hippocampal formations and the posterior cingulate region, with NAA/Cr proving to be the best predictor for AD. No correlation between hippocampal volumes and the results of 1H MRS in patients with AD was observed.

## Abstract (pt)

Estudos anteriores demostraram redução do volume hipocampal, redução da concentração de N- Acetilaspartato (NAA) e aumento da concentração de mio-inositol (mI) em pacientes com doença de Alzheimer (DA). O objetivo deste trabalho foi de avaliar os volumes hipocampais de pacientes com DA e correlacioná-los com as alterações metabólicas detectadas pela espectroscopia de próton das formações hipocampais e da região do cíngulo posterior.

**Material e Métodos:**

22 pacientes com provável DA (18 leve, 4 moderada) e 14 controles sem sintomas cognitivos foram incluídos neste estudo . Medidas volumétricas hipocampais, espectroscopia de próton de voxel único das formações hipocampais e da região do cíngulo foram obtidos. As seguintes razões de metabólitos foram avaliadas NAA/Cr, mI/Cr, mI/NAA. Análise estatística foi realizada para detectar as diferenças e correlações entre estes parâmetros nos pacientes e nos controles.

**Resultados:**

Os volumes das formações hipocampais dos pacientes e dos controles não foram significativamente diferentes. Os resultados da espectroscopia de próton foram significativamente diferentes nas das formações hipocampais dos pacientes e controles (mI/Cr, mI/NAA) e na região do cíngulo posterior (NAA/Cr, mI/Cr, mI/NAA). O melhor indicador para DA foi a razão NAA/Cr na região do cíngulo posterior com sensibilidade de 0,899 e especificidade de 0,800. Não houve correlação entre os volumes das formações hipocampais e os resultados da espectroscopia de próton nos pacientes com DA.

**Conclusões:**

Os resultados da espectroscopia de próton foram significativamente diferentes entre pacientes e controles das formações hipocampais e na região do cíngulo posterior, sendo NAA/Cr o melhor indicador para DA. Não houve correlação entre os volumes das formações hipocampais e os resultados da espectroscopia de próton nos pacientes com DA.

Alzheimer’s disease (AD) is a progressive neurodegenerative disorder associated with disruption of neuronal function and gradual deterioration in cognition, function and behavior. It is the most common cause of dementia in the elderly and affects more than 30 million individuals worldwide. Age is the strongest risk factor, with the disease affecting approximately 8% of individuals over the age of 65 years and 30% over the age of 85 years in developed countries.^1^ In developing countries however, there is a higher prevalence in younger subjects, perhaps owing to low educational level and cognitive reserve.^2^ The progression of AD is gradual and the average patient lives 8-10 years after the onset of symptoms.^1^

Neuropathologic degenerative changes occur first in medial temporal lobe structures, extending to neocortical association areas and later affecting the primary neocortex.^3^ In AD, the hippocampus is one of the earliest of the medial temporal limbic structures involved. Hippocampal volume loss is found in AD patients and correlates with dementia severity and neuropathologic changes.^4,5^

However, hippocampal atrophy is not specific to AD also occurring in normal aging. It has been speculated that this atrophy might be a useful disease marker in the early stages of AD, when the disease is largely confined to the medial temporal limbic areas, and loses its sensitivity as the disease advances.^5,6^

Proton magnetic resonance spectroscopy (1H MRS) allows noninvasive and quantitative assessment of a variety of cerebral metabolites. In vivo 1H MRS studies have shown that the N-Acetylaspartate (NAA) concentration is decreased and that myo-inositol (mI) concentration is increased, in patients with AD.^7-11^ These alterations in metabolite levels correlate with the severity of AD histopathology, indicating that these measurements could be used as biomarkers for disease progression in clinical trials.^12^

Our purpose was to evaluate hippocampal volumes of patients with AD and of controls, and their correlation with metabolic changes detected by 1H MRS of hippocampi and the posterior cingulate region.

## Methods

### Subjects

This study was approved by the institutional review board and all subjects or their legal representatives, where applicable, signed a written informed consent term.

Twenty two patients with probable AD (18 with mild and 4 with moderate dementia) according to the criteria of the National Institute of Neurological and Communicative Disorders and Stroke and the Alzheimer’s Disease and Related Disorders Association (NINCDS-ADRDA) and to the Diagnostic and Statistical Manual, 3^rd^ ed., revised (DSM-III R), were included in this study. Laboratory work-up was performed in all participants to rule out other causes of dementia.

Fourteen elderly volunteers without cognitive symptoms comprised the control group.

The average age was 74.2 years for patients (66 to 79 years) and 72.5 years for controls (56 to 87 years). Educational level was higher among patients (mean=8.9 years for patients vs. 6.0 years for controls).

Only subjects with satisfactory MR exams were included in this study.

### MR and MRS image acquisition

All studies were performed on a 1.5 T GE - Horizon LX 8.3 machine (General Electric Medical Systems, Milwaukee, WI, USA).

The single-voxel 1H MRS studies were performed with Point-Resolved Spectroscopy (PRESS) Pulse Sequence with an echo time (TE) of 35 milliseconds (ms) and repetition time (TR) of 1500ms.

In the posterior cingulate region, an 8cm^3^ voxel was prescribed on a midsagittal T1-weighted image including the right and left posterior cingulate and inferior precuneate region. In the hippocampal formation, an average 6cm^3^ voxel was prescribed, with 1cm thickness and variable width and length according to hippocampal dimensions (average of 1.5 x 4cm).

The 1H-MRS parameters analyzed in this study were NAA/Cr, mI/Cr and mI/NAA ratios, by post-processing with Magnetic Resonance User Interface (MRUI), Java version.

Hippocampal volumetric measurements were obtained from an oblique coronal plane from T1-weighted three-dimensional volumetric Spoiled Gradient Recalled Echo sequence (TE of 5 ms, TR of 11 ms, matrix of 320 x 192, flip angle of 10º, thickness of 1.6mm) independently by two trained radiologists. The anterior landmark of the hippocampal formation was the uncal sulcus and the posterior landmark was the crura of the fornices lift away from the hippocampal tail.^13^ Image processing was performed with Osirix Imaging Software for MacOS X. Region of interest boundaries were manually traced with a mouse-driven cursor. Once the outline of hippocampal formation had been defined, a slice area was calculated. The total volume of the structure was then calculated by multiplying the summed total area by slice thickness. The region of interest (hippocampal formation) was normalized for intersubject variation in head size by dividing by the total intracranial volume in each case.

### Statistical analysis

The T-test and Mann-Whitney test were used to determine differences between patients and controls, and Pearson’s correlation test was used to calculate correlation between volumetric measurements and metabolic ratios. A p value < 0.05 was considered statistically significant. The Bland & Altman test was used to determine agreement between the readers.

The sensitivity and specificity of 1H-MRS ratios were obtained for every observed value and represented as receiver operating characteristic (ROC) curves. ROC curves were generated by calculating the sensitivity and specificity of each observed predictor value and plotting one minus specificity against sensitivity. The area under the ROC curve was used to assess the diagnostic accuracy of 1H-MRS ratios in patients with AD.

## Results

The volume of each hippocampal formation was divided by the total intracranial volume.

The relative average volume of the right hippocampal formation was 0.00107 (±0.00030) cm^3^ in patients and 0.00120 (±0.00030) cm^3^ in controls. The relative average volume of the left hippocampal formation was 0.00103 (±0.00029) cm^3^ in patients and 0.00120 (±0.00027) cm^3^ in controls. The relative average volume of both hippocampal formations was 0.00105 (±0.00029) cm^3^ in patients and 0.00120 (±0.00027) cm^3^ in controls.

The left hippocampal formation relative volume of patients tended to be smaller than that of controls. The relative volumes of the right hippocampal formation, relative mean volume of both hippocampal formations combined, were not significantly different between patients and controls (Table 1).

**Table 1:** Relative volumes of right, left hippocampal formation and mean relative volumes of hippocampal formations in cm^3^.

	Hippocampal formation		
	Right	Left	Mean Right and Left
AD			
Mean	0.00107	0.00103	0.00105
SD	0.00030	0.00029	0.00029
Controls			
Mean	0.00120	0.00120	0.00120
SD	0.00030	0.00027	0.00027
Mann-Whitney	-1.525	-1.915	-1.590
p-value MW	0.133	0.057	0.116

AD, Alzheimer's disease; SD, standard deviation.

With regard to the metabolic ratios changes, significantly lower NAA/Cr and higher mI/Cr and mI/NAA ratios were detected in the posterior cingulate region of patients compared to controls (Table 2).

**Table 2:** NAA/Cr, mI/Cr and mI/NAA ratios in posterior cingulate region of patients with AD.

	Posterior cingulate region		
	NAA/Cr	mI/Cr	mI/NAA
AD			
Mean	1.632	0.830	.511
SD	0.088	0.115	00.075
Controls			
Mean	1.739	0.751	0.431
SD	0.098	0.068	0.035
Mann-Whitney	-3.041	-2.832	-3.935
p-value MW	0.002	0.004	<0.001

AD, Alzheimer's disease; SD, standard deviation; NAA/Cr, N-acetyl-aspartate/creatine; mI/Cr, myo-inositol/creatine; mI/NAA, myo-inositol/N-acetyl-aspartate.

There was an increase in mI/Cr ratio in the right hippocampal formation and an increase in mI/Cr and mI/NAA ratios in the left hippocampal formation. When both sides were combined, a significant increase in mI/Cr and mI/NAA ratios in the AD group was detected (Table 3).

**Table 3:** NAA/Cr, mI/Cr and mI/NAA ratios in right and left hippocampal formation and in both hippocampal formations.

	Right hippocampal formation				Left hippocampal formation				Mean right and left hippocampal formations		
	NAA/Cr	mI/Cr	mI/NAA		NAA/Cr	mI/Cr	mI/NAA		NAA/Cr	mI/Cr	mI/NAA
AD											
Mean	1.331	1.147	0.876		1.340	1.157	0.837		1.371	1.165	0.863
SD	0.176	0.112	0.135		0.363	0.303	0.239		0.177	0.133	0.137
Controls											
Mean	1.460	1.057	0.727		1.413	0.992	0.667		1.437	1.022	0.698
SD	0.144	0.137	0.089		0.337	0.147	0.104		0.195	0.133	0.092
Mann-Whitney	-1.887	-1.725	-3.085		-0.599	-3.201	-3.540		-0.811	-2.953	-3.317
p-value MW	0.059	0.085	0.001		0.567	0.001	<0.001		0.432	0.002	0.001

AD, Alzheimer's disease; SD, standard deviation; NAA/Cr, N-acetyl-aspartate/creatine; mI/Cr, myo-inositol/creatine; mI/NAA, myo-inositol/N-acetyl-aspartate.

The linear correlation between the normalized hippocampal formation volume and spectroscopy was not statistically significant. However, there was a trend towards a positive correlation between left hippocampal formation volume and NAA/Cr ratio.

The best predictor of AD was NAA/Cr in the posterior cingulate region. The area under the ROC curve was 0.828, with slightly higher sensitivity and specificity (0.899 and 0.800, respectively) at the optimal cutoff point (1.7) of NAA/Cr, compared to other ratios in the hippocampal formation.

## Discussion

Although hippocampal atrophy occurs at the initial stages of AD, hippocampal volume loss is also found in normal aging. Therefore, hippocampal atrophy can be helpful in diagnosing AD in younger patients but loses sensitivity with aging, when AD-related volume loss overlaps with normal aging volume loss.^6^

Neuropathologic studies in patients with AD have demonstrated neuritic plaques and neurofibrillary lesions, accompanied by neuron loss and gliosis, initially in the entorhinal cortex and hippocampus.^16^ Although some studies have reported differences between AD patients and healthy elderly subjects,^17^ other studies have shown an overlap between these groups, in terms of the amount of neurons that are replaced by glial cells.^18,19^ This could explain why no statistically significant difference in hippocampal volumes was detected between patients and controls in this study.

The hippocampal formation volume measurements can be obtained by manual or automatic methods. A wide range of hippocampal volumes were detected in several studies, even among controls, which likely stemmed from differences in hippocampal boundary criteria and the software used.^20^ Although manual volumetric measurement is considered the gold standard, it is labor intensive, with inter and intra-observer variability, while an automatic volumetric measurement method can be time saving and observer independent.^21^ The small volume of the hippocampal formations found in our study, compared to other papers, could be related to the above-mentioned variables.

The NAA/Cr, mI/Cr and mI/NAA ratios in the posterior cingulate region, and mI/Cr and mI/NAA ratios in the hippocampal formations, differed significantly between patients and controls. The mI/NAA ratio, which combines the two most affected metabolites in AD, is the most important ratio for the diagnosis of this disease as mI concentration increases due to gliosis while NAA level decreases as a result of loss of neuronal components, neuronal function disruption and neurofibrillary abnormality.^11,15^ Our study showed similar results, although the best predictor in the present sample was the NAA/Cr ratio.

The NAA/Cr ratio in the posterior cingulate region was able to discriminate controls from patients, proving slightly superior to the ml/NAA ratio in the posterior cingulate region and hippocampal formations. This finding could be explained by the fact that spectra in the posterior cingulate region is associated with better homogenization of the magnetic field, with less artifacts than in the hippocampal formations, although it is known that neuropathologic changes take place first in the medial temporal lobe structures.^3^

The lack of correlation between hippocampal volume loss and metabolic changes either in the hippocampal formations or the posterior cingulate region may be related to the low specificity of hippocampal atrophy in elderly patients, atrophy which may also be found in other causes of dementia.^5^ Other studies however have shown contrasting results.^22^ This lack of correlation may also have stemmed from the small number of individuals enrolled in this study, influencing the results. This finding could also indicate that changes in metabolite ratios may occur before hippocampal atrophy, with worsening ratios as the disease progresses.

In conclusion, the 1H MRS results differed significantly between patients and controls in the hippocampal formation and posterior cingulate region, but no correlation between hippocampal volumes and 1H MRS results was observed in patients with AD.
